# Implementing reduced-protein diets for broiler chickens in emerging economies: supplementation with only the first three limiting amino acids

**DOI:** 10.1007/s11250-025-04396-6

**Published:** 2025-03-31

**Authors:** Damilola U. Kareem, Adeola V. Adegoke, Adedoyin T. Amos, Emmanuel A. Adeyeye, Olubukola P. A. Idowu, Lateefat M. Akande, Quadri O. Abdulgafar, Adebukola T. Orbugh, Ayobami T. Aboderin, Abimbola O. Oso, Olusegun M. O. Idowu

**Affiliations:** 1 Department of Animal Nutrition, College of Animal Science and Livestock Production, Federal University of Agriculture, P.M.B, Abeokuta, 2240 Nigeria; 2 Poultry Research Foundation, The University of Sydney, Camden Campus, 425 Werombi Road, Brownlow Hill, 2570 NSW Australia; 3 Department of Animal Production and Health, College of Animal Science and Livestock Production, Federal University of Agriculture, P.M.B 2240, Abeokuta, Nigeria; 4 Agricultural and Environmental Science Institute, Federal University of Mato Grosso, Sinop, Mato Grosso Brazil; 5 Department of Animal Science, Mcgill University, 21111 Lakeshore, Sainte-Anne-de-Bellevue, QCH9X3V9 Canada

**Keywords:** Environment, Low-income economies, Low protein, Nitrogen emission, Poultry, Sustainability

## Abstract

This study evaluated the effects of reduced-protein diets supplemented with only the first three limiting amino acids (AAs); methionine, lysine, and threonine, on broiler performance, profitability, and nitrogen (N) utilization. Two hundred Cobb-500 broiler chicks were assigned to four dietary treatments in a Completely Randomized Design: a control diet and three reduced crude protein (CP) diets (-1PP, -2PP, -3PP). Diets contained CP levels of 21%, 20%, 19%, 18% during grower (14–28d) and 19.5%, 18.5%, 17.5%, 16.5% during finisher (28–42d) phases. Broilers on up to 2% CP reduction exhibited similar (*P < *0.05) or superior growth performance and feed conversion ratio compared to 3% CP reduction. Gross margin was highest (*P < *0.05) with up to 2% CP reduction but declined with further reduction. Carcass traits and breast yield decreased (*P < *0.05) with CP reduction, though meat quality was unaffected. N intake and retention decreased (*P < *0.05), while excretion and other N utilization parameters were unchanged. Nutrient digestibility remained consistent, but apparent metabolizable energy increased (*P < *0.05) as dietary CP decreased. These findings indicate that reducing dietary CP by up to 2%, supplemented with the first three limiting amino acids, maintains growth performance, profitability, and meat quality. This approach also reduces environmental N emissions and offers a cost-effective strategy for developing economies compared to reduced-protein diets with additional amino acid supplementation.

## Introduction

The global poultry industry faces increasing pressure to enhance production efficiency while addressing environmental sustainability and economic viability (Gržinić et al. 2023; Rodić et al. 2011). One promising strategy is the implementation of reduced protein diets for poultry (Woyengo et al. 2023), which has been shown to support similar growth performance up to upward of 3% reduction levels in maize-based diets (Chrystal et al. 2021, 2020b; Greenhalgh et al. 2020; Maynard et al. 2021) while minimizing nitrogen excretion (Alfonso-Avila et al. 2022; Lambert et al. 2022). Reducing dietary protein in broiler diets often requires augmenting the limiting amino acids with supplementation of crystalline amino acids (AA), which is usually outside the commonly supplemented first three limiting amino acids in poultry. The need to supplement these other amino acids, which are often more expensive, may increase the feed cost. Although the historical decline in the prices of the commonly supplemented AAs (methionine, lysine and threonine) suggests that the expensive AAs may become more affordable with increased demand in the future (Liu et al. 2021). Most of the studies on reduced protein has been in the developed world where despite the high cost of procuring the AAs, it is still accessible. Same cannot be said for low-income economies.

Poultry production has been reported to be an integral part of smallholder agriculture in the developing world otherwise termed emerging economies or low-income countries, and this has a multidimensional contribution to the livelihood of both rural and urban households (Birhanu et al. 2023). Since poultry serves as a vital source of protein and income in these regions, it is pertinent to equally adopt strategies that mitigate the detrimental impacts of the livestock sector on the environment, one of which is the adoption of reduced protein diets in broiler diets. Besides, OECD/FAO (2024) projects that Africa will contribute to the strongest growth in meat-related greenhouse gas emissions by 2033, followed by Asia and Latin America. Advocating for the implementation of reduced protein diets in low-income countries is therefore essential, not only for improving poultry production but also for promoting environmental sustainability and enhancing food security.

Since reduced protein maize-based diets support optimal growth performance, even if dietary protein is reduced up to 3%, but requires supplementing with AAs other than the first three limiting in poultry, which can be expensive, there is a need to identify the reduction level that supports optimal production performance when only the three limiting AAs (methionine, lysine and threonine) are supplemented—implementation of which will be suitable to developing economies. The current study therefore aimed to show that reduced-protein diets can be implemented in developing nations by evaluating its impacts when supplemented with only the first three limiting amino acids (methionine, lysine, and threonine).

## Materials and methods

### Research location

This study was carried out at the Poultry Unit of the Federal University of Agriculture, Abeokuta (FUNAAB), Ogun State, Nigeria.

### Experimental birds and management

A total of 200 one-day-old Cobb-500 broiler chicks (as-hatched) weighing 38 g ± 0.5 were obtained from a commercial hatchery. On arrival, the birds were housed together in a pen containing wood shavings as litter, drinkers, and tubular feeders. The chicks were brooded for 14 days. On d 14 post-hatch, the birds were individually weighed and randomly distributed into four dietary treatments of five replicates each, making a total of 20 experimental units (pens) with 10 birds each that were equalized on a weight basis per pen. Each pen had a dimension of 1.1 × 1.0 m, and a stocking density of approximately 10 birds/m^2^. Birds were given 12 h ad libitum access to feed and water throughout the experimental period. They were managed according to the Cobb-Vantress (2021) guideline.

### Experimental diets

The birds were fed a standard starter diet that met the guideline recommendation of Cobb-Vantress (2022) from 0 to 14 d post-hatch. On d14 post-hatch, the birds were randomly allotted to four isocaloric (AME; kcal/kg) experimental diets. The feeds were formulated to contain four protein levels (Control; −1PP [control minus 1 percentual point CP reduction]; −2PP [control minus 2 percentual point CP reduction]; and −3PP [control minus 3 percentual point CP reduction]). All other nutrients in the experimental diets met or exceeded the Cobb-Vantress (2022) nutrient specification guide. A two-phase feeding strategy was employed, so that the treatments had varying protein contents but similar other nutrient profiles at the grower (14 – 28 d) and finisher (28 – 42 d) phases. The control, −1PP, −2PP and −3PP diets were formulated with targeted levels of 21, 20, 19, and 18% CP during the grower phase; and 19.5, 18.5, 17.5, and 16.5% CP during the finisher phase, respectively. Only methionine, lysine, and threonine were supplemented in the diets—no other amino acids were supplemented. Composition of the experimental diets is presented in Table 1.

**Table 1:** Gross composition of diets

	Common starter	Grower (14 – 28 d)				Finisher (28 – 42 d)			
Ingredients (%)		Control	−1PP ^1^	−2PP	−3PP	Control	−1PP	−2PP	−3PP
Maize, grain	51.79	52.28	52.52	54.07	55.62	56.78	58.33	59.89	61.47
PKO	3.74	4.66	5.00	5.00	5.00	5.00	5.00	5.00	5.00
Wheat offals	0.00	2.71	5.43	7.03	8.63	1.80	3.40	4.94	6.46
Fish meal	1.37	0.00	0.00	0.00	0.00	0.00	0.00	0.00	0.00
Soybean meal	39.94	37.23	33.73	30.39	27.05	33.47	30.14	26.81	23.50
Bone meal	1.29	1.20	1.19	1.17	1.15	1.05	1.03	1.02	1.00
Limestone	0.64	0.69	0.73	0.77	0.82	0.73	0.77	0.82	0.86
Salt (NaCl)	0.31	0.24	0.21	0.18	0.15	0.24	0.21	0.14	0.05
Premix^2^	0.25	0.25	0.25	0.25	0.25	0.25	0.25	0.25	0.25
DL-Methionine (99%)^3^	0.33	0.31	0.34	0.36	0.38	0.28	0.31	0.33	0.35
L-Lysine HCl (78%)	0.12	0.11	0.20	0.29	0.38	0.09	0.19	0.28	0.37
L-Threonine (98.5%)	0.00	0.04	0.07	0.11	0.15	0.00	0.04	0.08	0.12
Choline chloride (60%)	0.10	0.10	0.10	0.10	0.10	0.10	0.10	0.10	0.10
Sodium bicarbonate	0.12	0.19	0.24	0.28	0.33	0.19	0.24	0.34	0.47
Total	100.00	100.00	100.00	100.00	100.00	100.00	100.00	100.00	100.00
Total NBAA	0.42	0.44	0.57	0.70	0.83	0.35	0.50	0.63	0.76
Calculated nutrient composition (%, unless otherwise stated)									
Crude Protein	22.50	21.00	20.00	19.00	18.00	19.50	18.50	17.50	16.50
AME (MJ/kg)	12.45	12.55	12.55	12.55	12.55	12.76	12.76	12.76	12.76
AME (kcal/kg)	2975	3000	3000	3000	3000	3050	3050	3050	3050
Net Energy (MJ/kg)^4^	11.74	11.87	11.88	11.89	11.90	12.14	12.15	12.16	12.17
Net Energy (kcal/kg)	2805	2837	2839	2842	2844	2900	2903	2906	2909
Crude Fibre	3.75	3.82	3.87	3.87	3.87	3.67	3.67	3.67	3.67
Neutral Detergent Fibre	11.61	12.73	13.72	14.31	14.90	12.33	12.92	13.48	14.04
Ether Extract	7.69	8.50	8.79	8.76	8.72	8.82	8.79	8.76	8.72
Ash	3.67	3.52	3.72	3.77	3.82	3.18	3.23	3.27	3.32
Calcium	0.90	0.80	0.80	0.80	0.80	0.75	0.75	0.75	0.75
Available Phosphorus	0.45	0.40	0.40	0.40	0.40	0.38	0.38	0.38	0.38
Sodium	0.18	0.16	0.16	0.16	0.16	0.16	0.16	0.16	0.16
Chlorine	0.29	0.23	0.23	0.23	0.23	0.23	0.23	0.21	0.17
Potassium	0.91	0.88	0.85	0.81	0.77	0.81	0.78	0.74	0.70
Digestible Lysine	1.26	1.16	1.16	1.16	1.16	1.06	1.06	1.06	1.06
Digestible Methionine	0.64	0.60	0.61	0.62	0.63	0.55	0.56	0.57	0.58
Digestible Met + Cys	0.94	0.88	0.88	0.88	0.88	0.82	0.82	0.82	0.82
Digestible Cysteine	0.30	0.28	0.27	0.26	0.25	0.27	0.26	0.25	0.24
Digestible Threonine	0.80	0.78	0.78	0.78	0.78	0.70	0.70	0.70	0.70
Digestible Glycine	0.86	0.77	0.73	0.69	0.64	0.72	0.67	0.63	0.59
Digestible Serine	1.07	1.00	0.94	0.88	0.82	0.93	0.87	0.82	0.76
Digestible Gly + Ser	1.62	1.48	1.40	1.32	1.23	1.38	1.30	1.21	1.13
DEB (mEq/kg)	230	230	221	212	202	214	204	200	200

^1^PP: Percentage point

^2^Provided per kg of vitamin/mineral premix: Folic Acid (min) 1600 mg; Vitamin B5—Pantothenic acid (min) 24.96 g; Biotin (min) 80 mg; Vitamin B5—Pantothenic acid (min) 24.96 g; Biotin (min) 80 mg; Butyl hydroxide toluene 100 mg; Niacin (min) 67.20 g; Selenium (min) 600 mg; Vitamin A (min) 13,440,000 UI; Vitamin B1 (min) 3492 mg; Vitamin B12 (min) 19,200 mcg; Vitamin B2 (min) 9600 mg; Vitamin B6 (min) 4992 mg; Vitamin D3 (min) 3,200,000 UI; Vitamin K3 (min) 2880 mg. Provided per kg of mineral premix: Copper (min) 15 g; Iron (min) 90 g; Iodine (min) 1500 mg; Manganese (min) 150 g; Zinc (min) 140 g

^3^ Purities (%) of non-bound amino acids are included in parentheses

^4^Calculated using the equation of Tay-Zar et al. (2024)

***PKO*** Palm kernel oil, ***DEB*** Dietary electrolyte balance, ***NBAA*** Non-bound amino acid

The dietary electrolyte balance (DEB) of the experimental diets was maintained to a minimum level of 200 mEq/kg as suggested by Arantes et al. (2013) and was calculated as DEB = Na + K – Cl while the net energy was calculated according to the equation of Tay-Zar et al. (2024), as shown below.

$$\text{NE }(\text{MJ}/\text{kg})=(0.815*\text{AME}(\text{MJ}/\text{kg})) -\left(0.026*\text{\%CP}\right)+(0.02*\text{\%EE})-(0.024*\text{NDF })$$

where, DEB = Dietary electrolyte balance, Na = Sodium, K = Sodium, Cl = Chlorine, NE = Net energy, AME = Apparent metabolizable energy, CP = Crude protein, EE = Ether extract, NDF = Neutral detergent fibre.

### Data collection

#### Growth performance and cost analyses

Data were recorded for body weight (g/b), weight gain (g/b*d), feed intake (g/b*d). The feed conversion ratio (FCR) was calculated from the values of weight gain and feed intake and was corrected for mortality.

The feeding cost variables: feed price (USD/tonne), feeding cost (USD/b), feeding cost per kg weight gain (FC/kgWG), and feeding cost per kg BW of the birds were calculated following the procedure reported in Kareem et al. (2022). The overall (cumulative) feed price (FP)/tonne was calculated as an average of the FP/tonne of the three phases.

$$\mathrm{FP}=\frac{Total\mathit\;cost\mathit\;of\mathit\;feed}{Quantity\mathit\;of\mathit\;feed};\;\mathrm{FC}/\mathrm b=FI\mathit/b\mathit\ast FP\mathit;\mathit\;\mathrm{FC}/\mathrm{kgWG}=FCR\mathit\ast FP;\;\mathrm{FC}/\mathrm{kgBW}=BW\mathit\ast FP$$

where, FP – Feed price; FC/b – Feeding cost per bird; FC/kgWG – Feeding cost per kg weight gain; FC/kgBW – Feeding cost per kg body weight; BW – Body weight; WG – Weight gain.

The economic returns (income and gross margin) were calculated according to the procedure of Azevedo et al. (2021). The selling price per kg of processed chicken of N 2600 (1.619 USD) was obtained from the market during the experimental period (February 2024) for calculation of the economic return. The exchange rate during the experimental period was 1 USD = N 1606.32 (CBN 2024). The total expenses, income and gross margin per bird were calculated. If 70% of total expenses = feeding cost (FC/b) (Oladokun and Johnson 2012), then, 30% of total expenses = other costs, therefore, Total expenses = FC/b + other costs. The calculations employed are highlighted below.

$$\text{Expenses }=\text{Feeding Cost}/\text{b }+\text{other costs}/\text{b}$$

$$0.7*\text{expenses}=\text{Feed Cost}/\text{b}$$

$$0.3*\text{expenses}=\text{other costs}$$

$$\text{Revenue }\left(\text{USD}/\text{b}\right)=\text{ Price}/\text{ kg body weight }\left(\text{USD}\right) \times \text{body weight }(\text{kg})$$

$$\text{Gross margin }\left(\text{USD}/\text{b}\right)=\text{ Revenue }\left(\text{USD}\right)-\text{ Expenses }\left(\text{USD}\right)$$

### Carcass traits and meat antioxidant enzyme activities

#### Carcass traits

On 42 d post-hatch, two birds per replicate whose body weights are close to the average weight of the group were selected, fasted (to clear their gastrointestinal tract (GIT)) for 12 h, weighed and humanely slaughtered for carcass traits evaluation. The birds were skinned, eviscerated and their cut parts weighed and recorded. The head, neck, and shanks were removed, after which the carcasses were weighed to obtain the carcass weight which was used to calculate the carcass yield. The cut parts considered include the breast (without skin), neck, wings, thigh, and drumstick. The cut parts were reported relative to the carcass weight (without shanks, head, and neck), while the abdominal fat, internal organs and carcass yield was reported relative to the live weight, in percentages.

### Meat antioxidant enzymes activity measurement

A 10 g meat sample was collected from breast portion of the carcass, for the carcass analysis. The breast meat samples were subjected to analyses to determine the antioxidant enzyme activities; superoxide dismutase (SOD), catalase and malonaldehyde (MDA).

#### Catalase

Catalase activity was measured via the disappearance of H_2_O_2_ characterized by a decrease in absorbance at 240 nm according to a modified version of a method described by Aebi (1984). A 3 g sample was mixed with 25 mL of 50 mM phosphate buffer (pH 7.0 at 25 °C) using a homogenizer (Ultra-Turrax T25 basic, IkaWerke GmbH & Co., Staufen, Germany) for 15 s at 13,500 rpm. The mixture was centrifuged at 1,800 × g at 2 °C for 15 min. The supernatant of the mixture was taken and filtered through a Whatman filter paper No. 1. Then, 100 μL of filtered supernatant was mixed with 2.9 mL of 30 mM H_2_O_2_. The decrease in absorbance at 240 nm was recorded every 30 s for 3 min. The catalase activity was expressed as units/g sample.

#### Superoxide dismutase (SOD)

SOD activity was measured using a modified version of a pyrogallol autoxidation method described by Marklund and Marklund (1974). First, 3 g of sample was homogenized with cold (4 °C) phosphate buffer (pH 7.0 at 25 °C) using a homogenizer at 13,500 rpm for 30 s. Then, centrifugation was performed at 1,800 g for 15 min at 2 °C using a JA-20 rotor (Beckman Instruments, Inc., Palo Alto, CA, USA) in a J2-21 centrifuge (Beckman Instruments, Inc., USA). The supernatant was filtered through a Whatman filter paper No. 1. Then, 50 μL of filtrate was transferred to a crystal cuvette (light path: 1 cm) and mixed with 3.025 mL of 50 mM Tris-cacodylate-DTPA buffer (pH 8.2; 25 °C) and 50 μL of 24.8 mM pyrogallol. The optical density was recorded at 420 nm every 15 s for 2 min. The SOD activity was expressed as units/g (U/g).

### Meat malondialdehyde content

Lipid oxidation in meat was determined by measuring the thiobarbituric acid reactive substances (TBARS) value according to procedure outlined by Sinnhuber and Yu (1977). A 0.5 g ground sample was mixed with 3 drops of an antioxidant solution (3% BHA-54% propylene glycol-3% BHT-40% Tween 20), 3 mL of a thiobarbituric acid solution (1% 4,6-Dihydroxy-2-mercaptopyrimidine), and 17 mL of 25% trichloroacetic acid. The mixture was heated in a water bath at 100 °C for 30 min followed by cooling for 30 min. The mixture was centrifuged at 2,400 × g for 30 min. The absorbance value of the supernatant was measured at 532 nm using a spectrophotometer (UV-mini-1240, Shimadzu, Japan). The result was calculated as mg malonaldehyde/kg sample.

### Meat quality and sensory evaluation

#### Meat pH

The probe of a standardized hand-held pH meter (pH meter model 108A—ATC Hana) was inserted into the meat. Hitherto, the pH meter was calibrated in buffer 4 and 7.

#### Cooking loss

Left thigh from one carcass per replicate was selected to ascertain cooking loss. Meat samples were weighed, wrapped in separate air-tight polythene bag and cooked in a water bath at 70 °C for 30 min (Sanwo et al. 2012).

#### Refrigeration loss

Right thigh from one carcass per replicate was randomly selected for determination of refrigeration loss. The samples were weighed and labelled prior to refrigeration, re-weighed after 24 h of refrigeration.

#### Water absorptive power

Ten grams of meat from the breast muscle was cut out and soaked in 10 ml of distilled water for 40 min per replicate. Thereafter, samples were removed, reweighed. Difference in weight obtained was expressed as a percentage of the initial weight.

### Sensory evaluation

Cooked meat from the cooking loss procedure was used to evaluate sensory attributes of meat. Post-cooking, samples were allowed to cool under room temperature and served to ten (10) semi-trained panellists; all within an age group of 18 – 25 years. Panellists were presented with the coded samples to evaluate attributes that range from colour to overall acceptability. Panellists scored the 7-point hedonic scale of Peryam and Pilgrim (1957), which was slightly modified by Sanwo et al. (2013). Water was provided for the panellists to gargle between assessments to curtail carryover effect.

### Nitrogen utilisation, emission, digestibility and apparent metabolizable energy

Excreta samples were collected for three days at the end of the d 14 – 28 and d 28 – 42 post-hatch periods of the experiment using total collection technique. The excreta samples were bulked, homogenized and sub-sample taken at the end of the excreta collection. The excreta were weighed, and oven dried at 65^0^C until a constant weight was obtained, followed by grounding to a size that passed through a 2 mm sieve. Nutrients were determined from the feed and excreta samples. To determine the apparent digestibility coefficient of the feed, the difference between the nutrient content in the feed and the excreta sample was multiplied by 100.

$$Nutrient\;digestibility(\%)=\frac{Nutrient\;in\;excreta\;(gDM)-Nutrient\;intake}{Nutrient\;intake\;(gDM)}$$

Apparent metabolizable energy (AME) of the diet was calculated according to procedure adapted from Sakomura and Rostagno (2016).

$$AME\;(kcal/kgdiet)=\frac{\left(Feed\;intake\;(DM)\times{GE}_{diet}\right)-\left(Excreta\;output\;(DM)\times{GE}_{excreta}\right)}{Feed\;intake\;(DM)}$$

Nitrogen retention was calculated using the equation of Sakomura and Rostagno (2016) by deducting the nitrogen in excreta from nitrogen excreted.

$$Nitrogen\;intake=Nitrogen\;in\;feed\;(\%DM)\times Feed\;intake$$

$$Nitrogen\;excreted=Nitrogen\;in\;excreta\;\left(\%DM\right)\times Excreta\;voided$$

$$Nitrogen\;retention=Nitrogen\;intake-Nitrogen\;excretion$$

Nitrogen efficiency was calculated following the equation of Lambert et al. (2022).

$$Nitrogen\;efficiency\;\left(\%\right)=\frac{Nitrogen\;intake\;\left(g\right)-Nitrogen\;retention\;\left(g\right)}{Nitrogen\;intake}\times100$$

Nitrogen (N) emission was determined by calculating the ammonia (NH_3_) and nitrous oxide (N_2_O) emissions from the excreta. These were calculated based on N volatilization equations described by Lambert et al. (2022) to determine N_2_O emissions as follows:

$$N\;volatilization\;\left(g/d\right)=N\;excretion\;\left(g/d\right)\ast\left(5.04\ast feed\;CP\right)\ast38.92$$

$$NH3\;volatilization\;\left(g/d\right)=N\;volatilization\;\left(g/d\right)\ast59.9\%$$

### Statistical analysis

All the data obtained from this study were checked for normality and homoscedasticity of error at 5% level of probability. Outliers were removed only after checking for plausibility. Data (excluding sensory) was then analysed as a one-way ANOVA using the SPSS 28 statistical package (IBM Corp 2021). Sensory data was subjected to Linear Mixed Model, with the panellists’ effect ‘weighted’ using GLM procedure of SPSS 28. Means were compared using Tukey test at 5% level of probability.

## Results

### Growth performance

The growth performance of the broiler chickens is presented in Table 2. At the grower phase, body weight and weight gain of the birds were significantly reduced despite significantly similar feed intake recorded between the different CP levels. Birds fed reduced protein diet up to 2% had similar (*P < *0.05) final weight and weight gain to those fed control diet. The FCR was significantly better for birds fed control and 1% CP reduction diets. During the finisher phase, FCR and feed intake of the birds were not significantly impacted by the varying dietary CP levels; however, the body weight and weight gain were better and similar for birds fed with control up to 2% reduced CP diets compared to those fed 3% CP reduction. The cumulative growth performance (14 – 42 d post-hatch) result shows that the body weight, weight gain, feed intake and FCR were all significantly impacted by the dietary protein levels. The broiler chickens had similar (*P < *0.05) and better values of the aforementioned variables up to 2% reduced CP diets compared with the birds fed 3% reduced protein. It was also observed that reducing dietary protein led to a significant reduction in overall feed intake, especially with 3% reduced CP.

**Table 2:** Growth performance of broiler chickens fed reduced protein diets

	Crude protein reduction level (PP^1^)					
	Control	−1PP	−2PP	−3PP	SEM	*P*-value
***Grower (14 – 28 d post-hatch)***						
Initial weight (g/b)	265.00	265.00	265.00	265.00	0.73	1.000
Body weight (g/b)	1076.00^a^	1050.44^a^	1008.56^a^	904.78^b^	17.03	0.000
Weight gain (g/b*d)	57.93^a^	56.10^a^	53.11^a^	45.70^b^	1.22	0.000
Feed Intake (g/b*d)	49.35	50.05	50.86	46.17	0.68	0.061
FCR	0.85^a^	0.89^ab^	0.96^bc^	1.01^c^	0.02	0.001
***Finisher (28 – 42 d post-hatch)***^***2***^						
Body weight (g/b)	1853.30^a^	1839.87^a^	1835.71^a^	1755.07^b^	50.80	0.000
Weight gain (g/b*d)	60.24^a^	59.28^a^	58.98^a^	53.22^b^	3.63	0.006
Feed Intake (g/b*d)	109.75	114.42	118.99	118.84	4.19	0.102
FCR	1.83	1.93	2.05	2.27	0.13	0.059
***Cumulative (14 – 42 d post-hatch)***						
Initial weight (g/b)	265.00	265.00	265.00	265.00	0.77	1.000
Body weight (g/b)	1960.65^a^	1908.47^a^	1833.56^a^	1576.94^b^	41.67	0.000
Weight gain (g/b*d)	60.56^a^	58.70^a^	56.01^a^	48.86^b^	1.49	0.000
Feed Intake (g/b*d)	83.15^ab^	88.69^a^	84.46^ab^	75.91^b^	1.26	0.033
FCR	1.37^a^	1.43^a^	1.52^ab^	1.62^b^	0.03	0.004

^abc^Means with different superscripts across the same row are significantly (*P < *0.05) different

^1^Percentage point

^2^Initial weight was used as a covariate variable

FCR—Feed Conversion Ratio

### Economic returns

Table 3 presents the economic returns of broiler chickens fed reduced protein diets for the entire production cycle (0 – 42 d post-hatch). The feed price, feeding cost/b, feeding cost/kgBW and feeding cost/kgWG were significantly affected by the varying levels of CP in the broilers’ diets. The feed price (USD/tonne) was observed to decrease (*P < *0.05) as the level of dietary protein reduced, while the feeding cost/kgBW and feeding cost/kgWG of the birds increased. It was observed that the feeding cost/kgBW and feeding cost/kgWG of the birds were similar for birds fed control diets up to those fed with 2% reduced CP diet, suggesting the suitability for up to 2% CP reduction when only methionine, lysine and threonine are supplemented. Interestingly, profit (gross margin) realised from the birds reduces as the dietary protein reduced, and was higher and better (*P < *0.05) up to 2% reduced CP diet.

**Table 3:** Economic returns of broiler chickens fed reduced protein diets at 0–42 days post hatch

	Crude protein reduction level (PP^1^)					
	Control	−1PP	−2PP	−3PP	SEM	*p*-value
Cost benefit						
Feed Price (USD/tonne)	395.58^a^	394.79^b^	394.34^c^	394.29^d^	0.12	0.000
Feeding Cost/ bird (USD/b)	1.05^ab^	1.07^a^	1.08^a^	0.99^b^	0.02	0.045
Feeding Cost/kg BW (USD)	0.54^c^	0.56^bc^	0.59^ab^	0.63^a^	0.01	0.002
Feeding Cost/kg WG (USD)	0.62^b^	0.65^b^	0.69^ab^	0.75^a^	0.02	0.001
Expenses and gross margin						
Expenses (USD/b)	1.37^ab^	1.40^a^	1.40^a^	1.28^b^	0.03	0.045
Revenue (USD/b)	2.07^a^	2.07^a^	1.94^ab^	1.65^b^	0.08	0.004
Gross margin (USD/b)	0.71^a^	0.68^a^	0.54^ab^	0.34^b^	0.07	0.018

^a^^−^^d^Means with different superscripts across the same row are significantly (*P < *0.05) different

^1^Percentage point

*BW- Body weight; WG—Weight gain; USD – US Dollar*

### Carcass traits

Carcass traits of the broiler chickens presented in Table 4 revealed that the liveweight (g), dressed weight (g), carcass yield (%), breast yield of the broiler chickens reduced (*P < *0.05) as the dietary protein reduced, following the trend of the growth performance. The other cut parts (back, wings, drumstick, and thigh) of the broiler chickens were not impacted (*P < *0.05) by the dietary CP level. The relative gizzard weight was observed to increase (*P < *0.05) with reduced dietary protein level; however, other internal organs (including abdominal fat) were not affected by the dietary CP level.

**Table 4:** Carcass traits of broiler chickens fed reduced protein diet

	Crude protein reduction level (PP^1^)					
	Control	−1PP	−2PP	−3PP	SEM	*p*-value
Liveweight (g)	1922.30^a^	1898.90^a^	1773.60^a^	1562.60^b^	29.97	0.000
Dressed weight (g)	1281.60^a^	1279.10^a^	1196.38^a^	1019.40^b^	25.13	0.000
Carcass yield (%)^2^	66.32^a^	67.32^a^	66.27^a^	63.37^b^	0.43	0.003
***Cut parts***^***3***^						
Breast (%)	33.31^a^	34.08^a^	32.30^ab^	29.78^b^	0.47	0.004
Back (%)	21.06	21.11	21.33	21.64	0.24	0.831
Wings (%)	11.28	11.70	10.90	12.15	0.21	0.190
Drumstick (%)	15.79	15.08	15.52	16.13	0.25	0.513
Thigh (%)	16.46	16.30	15.85	16.04	0.24	0.837
***Internal organs***^***2***^						
Heart (%)	0.48	0.43	0.46	0.49	0.01	0.321
Whole intestine (%)	3.33	3.15	3.36	3.71	0.08	0.104
Whole gizzard (%)	1.89^b^	1.95^b^	2.14^ab^	2.48^a^	0.06	0.000
Proventriculus (%)	0.39	0.37	0.39	0.44	0.01	0.154
Liver (%)	1.63	1.53	1.66	1.59	0.03	0.495
Kidney (%)	0.41	0.45	0.41	0.48	0.03	0.739
Spleen (%)	0.06	0.06	0.06	0.07	0.00	0.620
Abdominal fat (%)	1.78	1.87	1.68	2.04	0.12	0.759

^ab^Means with different superscripts across the same row are significantly (*P < *0.05) different

^1^ Percentage point

^2^% of liveweight

^3^% of dressed/carcass weight

### Meat quality, meat antioxidant enzyme activities and sensory evaluation

Meat quality, meat antioxidant enzyme activities and sensory evaluation of the broiler chickens are presented in Table 5. Reducing crude protein in broiler chickens’ diets has no significant effect on meat quality variables (water absorptive power, cooking loss, refrigeration loss, and meat pH) as all meat from the birds fed different CP levels present similar results. The meat antioxidant enzyme activities (malondialdehyde, catalase, superoxide dismutase) and acceptability depicted by meat sensory evaluation were also observed not to be significantly different among the different reduced dietary protein levels.

**Table 5:** Meat enzymatic-antioxidant, physico-chemical and sensory profiles of broiler chickens fed reduced protein diet

Meat	Crude protein reduction level (PP^1^)					
	Control	−1PP	−2PP	−3PP	SEM	*p*-value
***Enzymatic antioxidants***						
Catalase (U/g)	2.29	2.05	2.19	2.13	0.39	0.170
Superoxide dismutase (U/g)	2.49	1.29	2.33	2.81	0.33	0.410
***Malondialdehyde content***						
TBARs (MDA mg/g)	2.99	3.43	2.05	3.20	0.28	0.346
***Physico-chemical indices***						
Meat pH	6.46	6.46	6.48	6.50	0.05	0.993
Water Absorptive Power (%)	3.39	2.92	2.75	2.55	0.50	0.952
Cooking loss (%)	8.13	7.64	8.03	11.72	0.74	0.157
Refrigeration loss (%)	2.50	2.16	1.46	2.33	0.22	0.363
***Sensory evaluation***^***2***^						
Colour	6.10 (6)	6.06 (6)	6.08 (6)	6.26 (6)	0.08	0.818
Juiciness	5.54 (6)	5.76 (6)	5.70 (6)	5.94 (6)	0.12	0.710
Meat flavour	5.92 (6)	6.06 (6)	5.70 (6)	5.80 (6)	0.08	0.371
Tenderness	6.32 (6)	5.86 (6)	6.20 (6)	6.04 (6)	0.11	0.512
Saltiness	5.90 (6)	5.64 (6)	5.78 (6)	6.12 (6)	0.10	0.410
Overall flavour	5.28 (5)	5.12 (5)	5.30 (5)	5.62 (6)	0.12	0.515
Overall Acceptability	6.14 (6)	5.88 (6)	5.80 (6)	5.84 (6)	0.08	0.430

^1^ Percentage point

^2^ Values in parentheses are reported as approximate values due to the wholeness of hedonic scales

TBARs: 2- thiobarbituric acid reactive substance value

MDA: malondialdehyde

### Nitrogen utilisation, emission, digestibility and apparent metabolizable energy

The nitrogen intake (Table 6) significantly increased as the dietary protein decreased at both the grower and finisher phases. The nitrogen retention significantly decreased with reduced dietary protein at the grower phase; however, no significant effect was observed during the finisher phase. Other nitrogen emission and utilisation parameters were not significantly affected by the dietary protein levels regardless of the phase.

**Table 6:** Nitrogen utilisation, emission, digestibility and apparent metabolizable energy of broiler chickens fed reduced protein diet

	26 – 28 d post-hatch						40 – 42 d post-hatch					
	Control	−1PP ^1^	−2PP	−3PP	SEM	*P*-value	Control	−1PP	−2PP	−3PP	SEM	*P*-value
***Nitrogen utilisation and emission***												
N Intake	2.43 ^a^	2.20 ^ab^	1.80^b^	1.6 ^b^	0.18	0.035	3.22^a^	2.99^ab^	2.55^ab^	1.96^b^	0.18	0.041
N Excretion	0.33	0.27	0.27	0.25	0.02	0.357	0.29	0.27	0.21	0.21	0.01	0.110
N Retention	2.10 ^a^	1.93 ^ab^	1.53 ^ab^	1.35 ^ab^	0.11	0.048	2.93	2.72	2.34	1.75	0.17	0.062
N Efficiency	0.86	0.88	0.84	0.83	0.01	0.451	0.91	0.91	0.91	0.89	0.01	0.780
N_2_O Volatilization (mg/d)	35.57	29.49	28.79	24.24	1.87	0.200	155.28	146.91	114.05	114.19	7.89	0.114
***Nutrient digestibility and apparent metabolizable energy***												
Crude protein Digestibility	84.43	89.05	87.04	85.77	0.81	0.213	89.99	88.60	89.97	91.75	0.71	0.506
Ether extract Digestibility	77.86	82.00	75.70	78.84	1.21	0.314	86.72	84.29	86.20	88.84	0.98	0.460
Crude fibre Digestibility	79.55	84.78	82.60	83.64	1.00	0.284	87.41	85.55	89.11	90.82	0.80	0.069
Energy Digestibility	80.50	86.27	85.30	85.15	1.01	0.157	89.35	87.77	90.33	92.63	0.77	0.161
AME (MJ/kg)	11.48^b^	12.75^b^	12.59^ab^	13.41^a^	0.21	0.002	12.86^d^	13.25^c^	13.50^b^	14.31^a^	0.13	0.000

^ab^Means with different superscripts across the same row are significantly (*P < *0.05) different

^1^ Percentage point

AME – Apparent metabolizable energy

While the nutrient digestibility of the broiler chickens was not affected by the dietary protein levels, the apparent metabolizable energy (AME) increased (*P < *0.05) as the dietary protein levels decreased at both starter and finisher phases.

## Discussion

In many developing countries, livestock ownership serves as a fundamental measure of household socio-economic well-being, with poultry standing out as the most common species reared. This livestock sector is integral to food supply chains and income streams, especially in rural and peri-urban settings (Birhanu et al. 2023). However, the need to mitigate greenhouse gas emissions from the livestock sector, especially the poultry sector, makes it pertinent to ensure raising chickens sustainably. Reducing dietary protein is one of the methods employed to ensure this (Liu et al. 2021; Woyengo et al. 2023); however, the practicality of its implementation in low-income nations is doubtful due to the need to supplement some expensive “not-usually supplemented” essential AAs to meet up the AA requirements of the birds. This necessitates the need to find a solution that works within the confines of the regularly supplemented AAs (methionine, lysine and threonine) while reducing the dietary CP levels. Since 3% CP reduction has been reported optimal when supplementing all limiting AAs, how low can the reduction go when only the conventional AAs (methionine, lysine and threonine) are supplemented remains a question begging to be answered – one that this study tackled.

The similar body weight, weight gain, and FCR observed for the birds up to 2% CP reduction are in line with existing results (Chrystal et al. 2021, 2020a, b; Kareem 2023; Maynard et al. 2021), although slightly lower than the 3% CP reduction reported by Chrystal et al. (2021). However, it is worthy of note that no other amino acids other than the conventional AAs were supplemented in all the diets. This shows that the extra AAs supplemented in the aforementioned studies were the main cause of the improvement in the birds’ performance; however, with the current costs of the other AAs, the improved performance may not elicit increased profit due to the extra cost of AAs, especially in developing economies. There is a dearth of literature on the economics of broiler chickens fed reduced protein diets. In the current study, the feed price (USD/tonne), albeit marginal, was observed to increase as the dietary CP level decreased. This could be due to the increased dietary AA (methionine, lysine, and threonine) supplementation. While the feeding cost per bird was similar and higher for birds fed with up to 2% reduced CP, the feeding cost per kg weight gain was lower and better for same birds compared to those fed with 3% reduced CP. This suggests the suitability for up to 2% CP reduction in broiler diets even if only methionine, lysine and threonine are supplemented. The profit recorded for the birds reduced as the dietary CP reduced, with a higher and better values recorded for birds fed with up to 2% reduced CP diet. Although CP reduction up to 3% resulted in a drastic reduction in gross margin, the lower expense could prove advantageous to the farmers in developing economies.

**Fig. 1:**
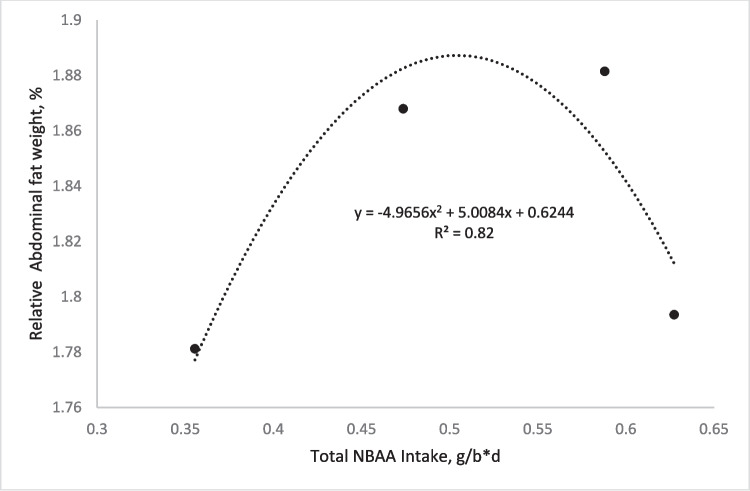
Graph showing the relationship between the relative abdominal fat weight and total NBAA intake as a function of the dietary CP level. NBAA: Non-bound amino acids

The carcass and breast yields follow the same pattern as the growth performance, suggesting that CP could be reduced in broiler diets up to 2% while supplementing with on the three conventional AAs (methionine, lysine, and threonine). It was surprising to find that the whole gizzard increased as the dietary CP decreased, the reason for which is quite unknown. However, we theorize that the dietary protein reduction which increased grain (corn) inclusion in the diet could have caused an increase in the percentage of large particles in the diets (mash), thus causing an increase in the gizzard's relative weight. It was also surprisingly observed that there was no significant difference in the abdominal fat pad, which is not in conformity with the report of some literature (Chrystal et al. 2021; Kareem 2023; Maynard et al. 2021; Woyengo et al. 2023). Perhaps the fact that the present study was not supplemented with other non-bound amino acids (NBAAs) reduced the total dietary NBAAs compared to what is usually recorded in other studies supplementing them in reduced protein diets. This caused a reduction in the NBAA intake and therefore could have allowed the birds to better utilize the excess starch usually associated with reduced protein diets, which could have impacted the birds’ glucose level and consequently fat deposition (Xiao & Guo 2022). The relationship between the relative abdominal fat weight and total NBAA intake as a function of the dietary CP level is presented in Fig. 1.

Meat quality of the birds in the present study was not impacted by the reducing dietary protein levels. The meat pH was similar for all the birds fed the different reduced protein diets, which is different from the result of Belloir et al. (2017). This could imply that the amount of glycogen in the birds’ muscle prior to slaughter was similar thereby reducing the rate of glycogen conversion into lactic acid after slaughter (Mir et al. 2017). Perhaps, this is one of the reasons for the similar abdominal fat recorded across the treatments, borne by the overall reduced total NBAA in the current study compared to other similar studies. Lipid oxidation is a complex process that profoundly influences the quality of poultry meat, flavour, nutritional profile, and shelf life (Domínguez et al. 2019). It is one of the challenges to the poultry meat processing industry because poultry meat is highly sensitive to oxidative reactions, which are responsible for rancidity in processed poultry products by inducing lipid oxidation (Nawaz and Zhang 2021). The increased fat deposition associated with reduced protein diets makes lipid oxidation determination important when reduced CP diets are fed to broilers. Malondialdehyde content of the birds, which is a measure of lipid peroxidation, was not significantly different across the different CP levels in the current study. This could indicate that reducing dietary protein in broiler diets does not predispose the meat post-slaughter to peroxidation.

While the nitrogen intake and retention reduced as the level of dietary protein decreased which is expected and in line with a previous study of Kareem (2023), the nitrogen excretion and consequently emission represented as N_2_O volatilization were surprisingly similar across the different reduced CP levels. Although the impact of the reduced CP on the N_2_O volatilization was not significant in the current study, a numerical reduction was still observed. This confirms the tendency of reduced protein diets to significantly reduce environmental impact of poultry production (Alfonso-Avila et al. 2022; Lambert et al. 2022). The apparent metabolizable energy (AME) increased as the dietary CP reduced. Although, the energy digestibility increase was not significant, the numerical increase observed with decreased dietary protein suggest that feeding low CP diets to broiler chickens has consequences on their energy metabolism which is usually deposited as fat (Lambert et al. 2022).

## Conclusion

The present study demonstrates that reducing dietary CP by up to 2% with supplementation of only the first three limiting amino acids (methionine, lysine and threonine) is possible. This can be adopted in developing nations due to its profitability relative to when other costly AAs are supplemented. Also, the study confirms the environmental reduction tendency of a reduced protein diet.

## Data Availability

Data generated from this study are available from the corresponding author on reasonable request.
